# Community health worker certification as a pathway to workforce development and career advancement: a qualitative study

**DOI:** 10.3389/frhs.2026.1902919

**Published:** 2026-10-05

**Authors:** Uchenna Benedine Okafor, Iyabo Obasanjo

**Affiliations:** 1 Center for Health Promotion and Disease Prevention, Edson College of Nursing and Health Innovation, Arizona State University, Phoenix, AZ, United States; 2 School of Graduate Studies, University of Maryland, Baltimore, MD, United States

**Keywords:** career advancement, CHW certification, community health workers, public health workforce, qualitative research, workforce development

## Abstract

**Background:** Community Health Workers (CHWs) play a vital role in bridging between healthcare systems and underserved communities. Although CHW certification has expanded across the United States, limited research has examined whether certification supports long-term career advancement and workforce development for CHWs themselves. This study examined how certified CHWs perceive the role of certification in shaping career trajectories and long-term workforce retention, with implications for CHW program design and public health workforce policy. A qualitative study using semi-structured interviews was conducted with 30 certified CHWs recruited from a database maintained by the Central Maryland Area Health Education Center (AHEC). Interviews were conducted virtually via Zoom and analyzed using Braun and Clarke's six-step reflexive thematic analysis framework. Five themes were identified: (1) CHW certification as a pathway to workforce development and career advancement; (2) CHW work as a calling rooted in service and advocacy; (3) CHWs as bridges between healthcare systems and communities; (4) barriers to certification and workforce integration; and (5) long-term commitment to public health and community impact. CHW certification serves a function beyond credentialing; it is a workforce development tool that supports career mobility, professional identity, and long-term engagement in public health. Addressing persistent barriers, including low wages, limited opportunities for advancement, and scope-of-practice confusion, is essential to sustaining a robust CHW workforce.

## Introduction

Community Health Workers (CHWs) have become an essential component of public health systems globally, particularly in underserved and low-income communities, where they improve healthcare access, provide culturally responsive support, and bridge gaps between healthcare systems and communities (1, 2). In the United States, CHW programs have been implemented to improve health outcomes among culturally, economically, and geographically disadvantaged populations (3). CHWs support chronic disease management, maternal and child health, mental health promotion, care coordination, and health education, while helping individuals navigate complex healthcare and social service systems (4–10). Beyond direct service provision, CHWs have contributed to community-engaged research and organizing efforts that strengthen trust between marginalized communities and healthcare institutions (11–14), and they played a critical role during the COVID-19 pandemic in disseminating accurate public health information and improving vaccination uptake (15).

The growing recognition of CHWs has intensified efforts to professionalize the workforce through formal training and certification programs. By 2022, at least 18 U.S. states had established state-recognized CHW certification programs (16), including Maryland, where this study was conducted. Although requirements vary by state, certification generally involves completing a state-approved training program, demonstrating core competencies, and, in some cases, passing a standardized examination. Certification is intended to standardize competencies, enhance workforce recognition, and support the integration of CHWs into healthcare and public health systems. Previous studies have demonstrated that CHW interventions are cost-effective and associated with improved health outcomes, including reduced stress, improved chronic disease management, and increased healthcare engagement (17–19). Despite these contributions, CHWs certified and non-certified alike continue to experience significant workforce challenges, including low wages, limited organizational support, job insecurity, and restricted career advancement opportunities (20, 21). Challenges that contribute to attrition and threaten the sustainability of CHW programs. While state-level certification programs have been associated with modest wage gains, their impact on job security and turnover remains limited (18), suggesting that certification alone may be insufficient to address the full spectrum of workforce challenges facing CHWs.

Employment is an important social determinant of health, with unemployment and precarious employment negatively affecting physical and mental well-being (22–24). Because CHW programs frequently recruit individuals from the same underserved communities they serve, CHWs often navigate the same socioeconomic and structural challenges as their clients (16). At the same time, as healthcare systems face workforce shortages driven by an ageing population and the lingering effects of the COVID-19 pandemic (25–28) the demand for a stable, well-supported CHW workforce has never been greater. CHWs represent a potentially important entry point into sustainable healthcare careers. CHWs regularly perform foundational healthcare tasks, such as care coordination, health education, patient navigation, and monitoring clinical indicators, which may prepare them for broader roles within healthcare systems (29). Yet, despite the growing policy interest in CHW certification, existing research has focused primarily on wage differentials and occupational turnover at the state policy level (18), leaving largely unexamined how certified CHWs themselves perceive certification as a pathway to long-term career advancement and professional integration within healthcare and public health systems.

Existing studies have largely focused on the effectiveness of CHWs in improving community health outcomes rather than on their own career trajectories and professional development. Research has consistently documented that CHWs face limited professional and career-building pathways, contributing to low wages, lack of advancement, and workforce instability (30). Efforts to address broader CHW workforce ecosystem challenges have emerged at the state level (31). Yet, how individual certified CHWs perceive and experience career advancement pathways remains underexplored.

This study is framed by the life course perspective on career development, which holds that career pathways are shaped by social institutions, prior life experiences, and structural conditions such as education, labor markets, and social roles (32). For CHWs, many of whom originate from historically underserved communities, these structural conditions may both constrain and create opportunities for upward career mobility. Maryland's integration of certified CHWs into medical homes and community-based care models (29) provides a particularly relevant context for examining how certification shapes employment trajectories and career aspirations. Therefore, this study explored how certified CHWs perceive the role of CHW certification in shaping their employment opportunities, career advancement, and long-term success within healthcare and public health sectors. Specifically, it examined participants’ prior employment experiences, current roles in the workforce, career aspirations, and perceptions of CHW certification's contribution to their professional development. This focus on certified CHWs was intentional, as the study sought to generate an in-depth understanding of the certification experience from those who have undergone the credentialing process.

## Method

### Study design

This qualitative study employed semi-structured interviews to explore certified CHWs’ perceptions of career development, employment trajectories, and the influence of CHW certification on workforce participation in the healthcare and public health sectors. The study used a reflexive thematic analysis approach to inductively generate themes from participants’ accounts.

### Participants and recruitment

Participants were recruited using purposive sampling from a database of 109 CHWs trained by the Central Maryland Area Health Education Center (AHEC) between 2017 and 2023, who subsequently obtained CHW certification through the state of Maryland. The database included participant names, email addresses, and training and certification dates. We sent an invitation email describing the study purpose to all individuals in the database. We enrolled the first 30 participants who responded and scheduled an interview. This corresponds with a response rate of approximately 27.5% (30 of the 109 CHWs invited). Because enrollment was limited to the first 30 respondents, participants may differ from non-respondents in ways that could bias findings toward a more positive view of certification as a pathway to advancement.

A sample of 30 was considered appropriate for qualitative interview research, consistent with evidence that thematic saturation in community health studies is typically achieved within 20–30 interviews (33, 34). Participants ranged in age from 24 to 76 years and represented diverse educational and professional backgrounds; Table 1 reports full demographic characteristics.

**Table 1:** Participants’ demographic characteristics (*N* = 30).

Variables	*n*
Age Group (years)	
20–29	8
30–39	5
40–49	2
50–59	9
60 years and above	5
Missing	1
Educational Level	
High School/GED	3
Associate degree	3
College/Some College	10
Bachelor's Degree	4
Master's Degree/Pursuing Master's	5
Undergraduate/Certificate	3
Missing	2
Employment Status	
Currently employed	22
Unemployed/Volunteer/Student	6
Missing	2
Annual Income/Salary Range	
Less than $30,000	5
$30,000–$49,999	8
$50,000–$69,999	10
$70,000 and above	3
Hourly/Weekly wage reported	3
Missing	1
Time to Certification After Training	
Immediately–1 month	11
2–3 months	8
4–12 months	5
More than 1 year	2
Missing	4
Number of Jobs Since Certification	
None	3
One job	13
Two jobs	5
Three or more jobs	2
Missing/Not reported	7

"College/Some College” indicates some college coursework completed without an associate or higher degree; “Undergraduate/Certificate” indicates completion of a non-degree undergraduate certificate program. Three participants reported income as an hourly or weekly wage rather than an annual figure; these were not converted to an annual-equivalent estimate because weekly/annual hours worked were not consistently recorded for these participants and were reported in their original units rather than assigned to an annual income bracket.

### Data collection

An interview guide comprising 14 semi-structured questions and six demographic items was developed to explore participants’ experiences with CHW certification, employment trajectories, current workforce roles, career aspirations, perceptions of career advancement opportunities, and the role of CHW training in professional development. UBO and IO, the primary authors, developed the guide by drawing on the CHW workforce-development literature reviewed in the Introduction and the study's life course theoretical framework, and refined it through pilot testing before data collection. The full interview guide is provided as Supplementary Material (Appendix A).

A Maryland-certified CHW who completed training through the Central Maryland AHEC program conducted all interviews. Before data collection, the interviewer received training from the first author on study objectives, interview procedures, and use of the interview guide. The use of a trained CHW as an interviewer was intentional, as shared professional identity is known to facilitate rapport and candor in qualitative research (43).

Interviews were conducted virtually via Zoom between February 18 and April 7, 2025, and lasted approximately 45–90 min on average. Participants provided verbally informed consent, including consent for audio recording, at the outset of each session. Interviews were video recorded to allow the interviewer to observe nonverbal cues, then electronically transcribed and reviewed for accuracy.

### Data analysis

Data was analyzed using reflexive thematic analysis guided by Braun and Clarke's six-step framework (35). The research team familiarized themselves with the data through repeated reading of transcripts. UBO and IO coded independently, each coding a subset of transcripts before comparing codes; they resolved divergent interpretations through discussion and consensus rather than a formal inter-rater reliability statistic, consistent with the collaborative, interpretive orientation of reflexive thematic analysis (35). Transcripts and codes were managed using qualitative software e.g., NVivo. Data saturation was assessed iteratively during analysis by reviewing transcripts and emerging codes from the 31 enrolled participants to determine whether additional interviews produced new themes or meaningfully expanded existing categories. Initial inductive codes were generated from participant responses and grouped into broader categories based on recurring patterns and shared meanings; these candidate categories were then iteratively refined through repeated comparison across transcripts and team discussion into the five final themes and associated subthemes reported below, capturing participants’ experiences with workforce development, professional identity, career mobility, barriers to advancement, and long-term career aspirations. Themes were reviewed for coherence and alignment with the study objectives, and representative participant quotations were retained to support thematic interpretation and enhance credibility. When a quotation is attributed to more than one participant, it indicates that those participants expressed similar views, and one representative verbatim quotation was selected for readability rather than presenting each similar statement separately.

### Ethical considerations and trustworthiness

The Institutional Review Board of the University of Maryland, Baltimore, reviewed this study and determined it to be exempt (HP-00113008). Participation was voluntary, and all participants provided informed consent prior to interviews. Recordings and transcripts were securely stored and accessible only to the research team. To establish trustworthiness, we employed several strategies. Participants were recruited from diverse professional and educational backgrounds to enable a broad range of perspectives. All interviews were video-recorded and transcribed verbatim; recordings were reviewed multiple times to verify transcription accuracy. We maintained an audit trail documenting methodological decisions, coding processes, theme development, and analytical reflections throughout.

Positionality statement: The first author (UBO), a public health researcher with prior scholarship on CHW job roles and experience training and working alongside CHWs, led transcription and data analysis. The second author (IO), a public health researcher and epidemiologist, conceptualized the study and managed the interview process. Both authors approached the data with an interest in CHW workforce equity and acknowledge that our professional and academic proximity to CHW work, including prior research on CHW job roles (2), may have shaped our interpretive lens, including a disposition toward foregrounding certification's benefits. We engaged in ongoing reflexive discussion throughout coding and theme development specifically to surface and interrogate this and other assumptions [R1-3].

Consistent with reflexive thematic analysis, the involvement of multiple researchers served not to verify coding agreement through a formal reliability statistic, but to enrich interpretation through dialogue: each coder independently reviewed transcripts and proposed codes, and the research team then met iteratively to compare coding decisions, discuss areas of divergent interpretation, and reach a shared, negotiated understanding of emerging codes and themes through discussion rather than majority vote. Researchers engaged in iterative discussion of codes, emerging themes, and analytic decisions throughout the process, a practice of collaborative reflexivity that strengthens analytical depth and transparency (35).

## Results

Participants’ ages ranged from 24 to 76 years. Most participants had a college-level education or higher (*n* = 22), and most were employed at the time of the interview (*n* = 22). Most lived with family members, spouses, or children (*n* = 21). Annual incomes ranged widely from less than $30,000 to over $70,000, with the largest cluster in the $50,000–$69,999 range (*n* = 10). Most participants received certification within three months of completing training (*n* = 19). In Maryland, certification is not automatic upon completing an accredited training program: CHWs must submit a separate application to the Maryland Department of Health, which conducts a completeness review (up to 30 calendar days) followed by a final determination (an additional 1–90 calendar days), a process that can take up to 120 days in total (42). The interval reported here therefore reflects this administrative certification timeline rather than variation in individual competency attainment. The largest share of participants had held one job since certification (*n* = 13). Nearly all participants (*n* = 28) reported that their CHW training helped them perform their current roles.

Thematic analysis identified five major themes and corresponding sub-themes related to the role of CHW certification in workforce development, career advancement, and long-term participation in healthcare and public health. These are summarized in Table 2.

**Table 2:** Summary of themes and sub-themes.

Theme	Sub-themes
1. CHW Certification as a Pathway to Workforce Development and Career Advancement	Professional legitimacy and workforce recognition Career mobility and advancement opportunities Transition into healthcare and public health roles Educational advancement and leadership aspirations
2. CHW Work as a Calling Rooted in Service and Advocacy	Passion for helping people Advocacy and community empowerment Emotional fulfilment and personal satisfaction Building trust and relationships within communities
3. CHWs as Bridges Between Healthcare Systems and Communities	Resource navigation and referrals Health education and community outreach Addressing social determinants of health Cultural understanding and active listening
4. Barriers to Certification and Workforce Integration	Delays in certification and practicum placement Workforce instability, low wages, and under-recognition Personal challenges affecting workforce participation
5. Long-Term Commitment to Public Health and Community Impact	Desire to continue working in healthcare/public health Mentorship and teaching aspirations Community leadership and entrepreneurship goals

Where a quotation below is attributed to more than one participant (e.g., “Participants 1, 6”), see Data Analysis for an explanation of this convention.

### Theme 1: CHW certification as a pathway to workforce development and career advancement

CHW certification emerged as an important mechanism for workforce development and career advancement. Participants described certification as strengthening professional legitimacy, enabling transitions into more stable healthcare and public health roles, and motivating further educational and leadership aspirations. This theme is central to the study's core research question: rather than functioning only as a credential, participants described certification as an active mechanism that opened doors to employment, further training, and a stronger sense of professional identity.

#### Professional legitimacy and workforce recognition

CHWs regarded certification as enhancing their credibility within healthcare and public health systems while strengthening professional identity and confidence. Participants described their CHW credentials as a foundational layer of knowledge upon which additional certifications and competencies were built:

“The CHW is foundational base knowledge while other certifications build on it. It gave me a clear understanding, depending on the community and population that I am working with.” (Participant 10)

“It is the first thing that you are being asked when you apply for a job.” (Participant 16)

Together, these accounts suggest that certification functioned less as a formality and more as a credibility signaling that participants actively invoked both internally, in how they understood their own expertise, and externally, in employer screening.

#### Career mobility and transition into healthcare and public health roles

Participants described how certification created tangible opportunities for employment, promotion, and transition into more stable positions. Several described clear upward trajectories from entry-level or temporary roles into supervisory, coordination, and specialist positions:

“When I got certification in 2018, I worked as an ED navigator and community health worker. One year later, I was transferred to another department as a family support specialist, where I conducted home visits. Then I became a service coordinator case manager, and after that, I became a supervisor for community health workers, which I am currently at.” (Participant 3)

“I have been with MedStar since my entire CHW journey, community health advocate 1, and I am currently a program coordinator.” (Participant 9)

These trajectories illustrate a concrete mechanism behind the “pathway” framing of this theme: certification did not merely coincide with career progression; participants described it as the credential that made each successive transition possible.

#### Educational advancement and leadership aspirations

Certification motivated many participants to pursue further education, specialized training, and leadership roles. Several expressed aspirations to obtain bachelor's, master's, or doctoral degrees in public health, social work, medicine, and healthcare administration:

“I am doing my Master’s in Public Health, then PhD in Public Health, then work in my company to make a change.” (Participant 14)

“I want to go back to school again to get a degree in the public health field… I would like to keep building myself and would like to be a consultant specialist one day.” (Participants 10, 13)

### Theme 2: CHW work as a calling rooted in service and advocacy

CHW work emerged as a calling deeply rooted in service, compassion, and advocacy. Participants consistently described their roles as extending well beyond employment, encompassing a commitment to helping vulnerable individuals, connecting communities to resources, and promoting community well-being. This theme captures the intrinsic motivation that sustained participants’ commitment to the work even where structural conditions were unfavorable.

#### Passion for helping people

Participants described helping others as a natural disposition rooted in compassion and personal commitment, frequently using the word “passion” to characterize their relationship to the work:

“I love helping people, I advocate, educate and connect them to resources, and this is what I do every day. I love it, and I am passionate about being a CHW… I love the smile on people’s faces after helping them” (Participants 1, 6)

“It is my calling to be a CHW. I always like to help and care for people. I have a lot of elderly people in my family who suffer from many illnesses, so I have been caring for them.” (Participants 10, 16)

#### Advocacy and community empowerment

CHWs described their advocacy as central to their professional identity, giving community members a voice, navigating systems on their behalf, and empowering individuals to make informed decisions about their health:

“One thing I learned about being a CHW is advocacy; you must learn how to advocate for your people.” (Participant 3)

“We need to do whatever it takes to make sure that people have basic, good-quality, affordable healthcare and that people are educated.” (Participant 15)

#### Emotional fulfilment and personal satisfaction

Witnessing positive changes in others’ lives generated a strong sense of purpose and satisfaction among participants. Many described emotional fulfilment as a key reward of CHW practice:

“After doing my job, I feel satisfied that I did my job… I love the smile on people’s faces after helping my community.” (Participants 1, 10)

“Being a CHW makes me feel good because I have the opportunity to spend time and connect with people.” (Participants 11, 12)

#### Building trust and relationships within communities

Trust-building was identified as foundational to effective CHW practice. Participants described how genuine relationships enabled deeper understanding of community needs and more effective service delivery:

“The knowledge that I have as a certified CHW… is the connection I have with people. It helps me to listen to people and encourage them to fight for themselves. Sometimes we don’t solve all problems, but the connections I have with people are something that I will cherish.” (Participants 4, 7)

“Understanding that part will open another door to connect with people… We don’t know what is behind the curtain. Understanding alone helps me to understand people and connect with them.” (Participants 11, 18) Across these subthemes, participants did not describe passion and professional advancement as competing motivations; rather, a sense of calling appears to have coexisted with, and in several accounts reinforced, the career-mobility gains.

### Theme 3: CHWs as bridges between healthcare systems and communities

A defining theme across the interviews was CHW's role as a bridge connecting underserved individuals to healthcare systems, resources, and information they could not easily access on their own. Participants described this bridging function across four domains: resource navigation, health education, addressing social determinants of health, and culturally responsive engagement. This theme links CHWs’ day-to-day bridging work to the study's broader workforce development because this expertise is difficult for conventional clinical roles to replicate, supporting CHW certification as a distinct professional pathway rather than a lesser substitute for clinical credentials.

#### Resource navigation and referrals

Participants described resource navigation as a central daily function, locating and connecting community members to social services, healthcare programs, and material support:

“CHW came into existence to bridge the gap between nurses and doctors and the people, and to make their jobs easier.” (Participant 1)

“Just being able to help people, being a resource connector, being able to advocate for them, and serving the underserved community in Baltimore.” (Participant 10)

#### Health education and community outreach

CHWs described active roles in educating community members about chronic disease management, preventive health, and system navigation, extending the reach of clinical providers into homes and communities:

“I advocate, educate and connect them to resources, and this is what I do every day.” (Participant 1)

“I love the flexibility, being able to engage with the community, the help and support that I rendered to the community.” (Participant 3)

#### Addressing social determinants of health

Participants emphasized that CHWs are uniquely positioned to identify and respond to social determinants, including housing, food insecurity, and financial instability, that clinical providers often overlook:

“Sometimes I feel like the screening we are doing is not enough. We see a lot of people that are helpless… asking them about their living situations, finances, healthcare, mortgage.” (Participant 1)

“The doctors we work with do not understand the challenges people face. But we, the CHWs, do understand the challenges people face daily.” (Participant 12)

#### Cultural understanding and active listening

Participants identified cultural competence and active listening as essential tools that distinguished CHW practice from conventional clinical interactions. Shared backgrounds and lived experience enabled CHWs to engage communities in ways that formal health providers often could not:

“I understand why I do what I do to connect with different individuals with different ethnicities. More knowledge and more power.” (Participant 11)

“Housing is a big component, and it depends on the need. Being from the community means I understand what people are facing.” (Participant 16)

### Theme 4: barriers to certification and workforce integration

Despite the positive outcomes associated with certification, participants identified significant barriers affecting both the certification process and subsequent workforce integration. These barriers encompassed administrative delays, structural workforce challenges, and personal life circumstances. This theme shows that the career-advancement benefits of certification described above were not universal or automatic; they were conditioned by administrative, financial, and personal circumstances that varied unevenly across participants.

#### Delays in certification and practicum placement

Several participants described difficulties securing practicum placements, completing required practice hours, and navigating administrative processes to obtain certification. Virtual training modalities during COVID-19 further complicated placement opportunities:

“Finding space to be accommodated in the workplace is difficult because the training was virtual, it was tough and difficult.” (Participants 6, 7)

“The person who was supposed to issue the certificate was not responding until the supervisor intervened, and finding a place to work for 40 h was challenging. I almost gave up.” (Participants 10, 13)

#### Workforce instability, low wages, and under-recognition

Participants consistently described concerns about low compensation, temporary contracts, limited opportunities for advancement, and inadequate recognition of the CHW role within healthcare systems. Several noted that their contributions were undervalued relative to their responsibilities:

“I feel like we are not appreciated enough. There isn’t much growth in community health worker roles. I know that CHWs still have a long way to go. I felt that we should have a cushion because there are no respect and regard for us.” (Participants 6, 16)

“I see a lot of people doing CHW jobs who do not have a certificate. I feel like COVID-19 has exposed a lot of things, and we still have a lot to do.” (Participants 10, 19)

#### Personal challenges affecting workforce participation

Illness, caregiving responsibilities, childcare demands, and other personal circumstances affected participants’ ability to complete certification requirements and maintain sustained workforce engagement:

“The only challenge for me was having respiratory health challenges, caring for elderly family members with illnesses, while navigating work.” (Participants 1, 4, 16)

“I was a nursing mom at the time… and later I left the job because I was diagnosed with liver cancer, stage 4, which has metastasized. I am currently going for chemotherapy now.” (Participants 12, 17)

These accounts suggest that the barriers identified here are not simply individual misfortunes but recurring structural features of the certification and employment pathway.

### Theme 5: long-term commitment to public health and community impact

Despite the barriers they described, participants expressed a strong and enduring commitment to public health and community service. Many articulated plans to deepen their impact through continued advocacy, mentorship, teaching, and community leadership. The participants’ aspirations to teach, mentor, and lead suggest that the CHW workforce is not only a service-delivery asset but a potential pipeline for public health leadership.

#### The desire to continue working in healthcare and public health

The overwhelming majority of participants expressed the intention to remain within healthcare and community-based sectors, driven by a sustained sense of purpose and service:

“Yes, I see myself continuing to work in public health. I feel like I can continue to advocate for and help people who cannot advocate for themselves. I am motivated to do that.” (Participants 1, 2, 3, 10)

“Because of my role as a CHW, it helps me to navigate and help the community. Listening and supporting people made me go and look up how to respond to people.” (Participant 8)

#### Mentorship and teaching aspirations

Several participants expressed strong interest in teaching CHW courses, guiding emerging professionals, and sharing their experiences to strengthen the pipeline of trained CHWs:

“I would like to continue to lead people behind me, empower them, and teach them. I have a drive to teach and lead other people. I would like training to teach CHW courses it is something that I love to do.” (Participants 1, 13, 15)

“The CHW helped me to understand who I am and what I need to live for, and the barriers I have within myself. I feel like I must advocate and teach other people.” (Participants 7, 11)

#### Community leadership and entrepreneurship goals

Several participants articulated ambitions beyond direct service delivery, including establishing community programs, leading organizations, and creating social enterprises to address persistent health and social inequities:

“I am looking forward to being an entrepreneur in the next two years. I feel like I have the network to help people with finding jobs, connecting people to community services, focusing on government contracts, procurements, and staffing.” (Participants 2, 3, 4)

“I want to open a resource center as a legacy center for people in need.” (Participant 6)

## Discussion

This study addressed a question the existing literature has not directly answered: how certified CHWs perceive and experience the consequences of certification for their career trajectories, professional identity, and long-term retention in Public Health. Prior research has examined CHW certification primarily through wage differentials and turnover rates at the state policy level (16, 18, 20), leaving largely unexamined the mechanisms through which certification shapes or fails to shape individual career pathways from the perspective of those who hold the credential. The findings address this gap. Certification emerged not merely as a professional credential but as an inflexion point in CHWs’ working lives: it opened employment opportunities, provided a foundation for stackable credentialing, cultivated a professional identity that sustained workforce commitment even under adverse structural conditions, and in several cases catalysed trajectories extending from entry-level community health work into supervisory, educational, and entrepreneurial roles.

However, these outcomes did not appear automatic; participants’ accounts suggest they were associated with institutional support, fair compensation, and genuine advancement infrastructure. Where participants described those conditions as absent, certification alone did not appear, in their accounts, sufficient to overcome the structural barriers they described as constraining CHW career advancement, a pattern that suggests, though this cross-sectional qualitative design cannot establish causality, that credentialing and workforce investment may need to be pursued together rather than sequentially.

The finding that certification created concrete pathways from entry-level and volunteer positions into coordination, supervisory, and specialist roles directly addresses a gap in the CHW workforce literature. While prior studies have called for clearer career advancement structures for CHWs (31, 36) and documented the absence of such pathways as a driver of attrition (20, 30), they have not examined how certification functions as the specific mechanism through which those pathways are accessed. The present study provides that evidence. Participants did not describe certification as a symbolic credential; they described it as the practical threshold that enabled employment transitions, signalled professional legitimacy to employers, and motivated investment in further education. This moves the conversation beyond identifying the need for career pathways to demonstrating, from CHWs’ own accounts, how certification can serve as an entry point into them.

Participants described CHW certification as a foundational credential upon which additional qualifications, mental health first aid, diabetes education, laboratory associate training, and formal degrees were systematically layered, consistent with the concept of stackable credentials in the workforce development literature. This credential stacking appeared to increase both employability and prospects for advancement, creating compounding professional returns that a single certification alone could not deliver. Importantly, however, these benefits accrued to those who could access complementary pathways, raising an equity concern that the data make visible without fully resolving. CHWs who faced persistent structural barriers, including low wages, caregiving responsibilities, and limited institutional support, were least positioned to pursue additional credentialing, meaning the upward mobility that stacking enables may systematically bypass those who need it most. CHW training programmes must therefore do more than signal the value of stackable credentials; they must actively facilitate equitable access.

The near-universal articulation of CHW work as a “calling” rooted in service and advocacy aligns with the broader qualitative literature on CHW identity and motivation (13, 37).

The present findings, however, extend this evidence in a critical direction. Intrinsic motivation appeared to sustain workforce commitment even in the face of low wages, job insecurity, and institutional under-recognition, not because participants viewed those conditions as acceptable, but because their sense of purpose ran deeper than the structural failures surrounding them. This finding demands a policy response, not simply a policy concern. When passion serves as a de facto substitute for adequate compensation and structural support, the cost of workforce sustainability is effectively externalised onto individual CHWs, many of whom already navigate significant socioeconomic burdens. The life course perspective makes this inequity explicit. For individuals whose early-life circumstances have already constrained their economic options, relying on intrinsic motivation as a retention strategy is not merely insufficient; it is a compounding injustice that health systems should stop depending on.

Participants described their bridging function in concrete terms: navigating resources, delivering health education, addressing social determinants of health, and engaging communities through shared cultural understanding and lived experience. What distinguishes this bridging role is not simply its scope but its mechanism: CHWs access communities through trust, shared identity, and lived proximity in ways that formal health providers, however well-intentioned, are structurally positioned to replicate only with difficulty (29). That this function continues to be performed amid significant workforce instability makes the bridging contribution more remarkable and the structural neglect indefensible. The barriers identified delays in certification, low wages, temporary contracts, and personal health and caregiving challenges are consistent with workforce instability documented in prior studies (16, 20, 38, 39). The COVID-19 pandemic brought these tensions into sharper relief: participants described how the shift to virtual training complicated practicum placement and the completion of required practice hours, while others observed that the pandemic exposed the extent to which individuals without formal certification were performing CHW roles. Rather than resolving these structural gaps, the pandemic made them impossible to ignore, reinforcing that workforce investment, including accessible certification pathways and stable employment, cannot be deferred or made contingent on crisis conditions.

Participants’ expressions of long-term commitment and aspirations to teach, lead, and establish community enterprises reveal that the CHW workforce is not only an asset for current service delivery but a pipeline for future public health leadership. Participants described concrete trajectories already underway: moving from entry-level roles to supervisory positions, pursuing graduate education, and pursuing entrepreneurial ventures. Realizing this potential requires that training programs move beyond credentialing to provide career counselling, mentorship, and professional development support that translate certification into sustained career momentum (36). The infrastructure for a more equitable public health leadership pipeline already exists within the CHW workforce; what is missing is the institutional will to invest in it.

Taken together, the findings support the life course framework's emphasis on social institutions’ role in shaping career trajectories (32). CHW certification functions as one such institutional mechanism, one that, in participants’ accounts, appears to expand career mobility when adequately resourced and supported, motivates further education, and anchors long-term commitment to public health service. For CHWs who originate from historically marginalised communities, the stakes extend beyond individual career outcomes: investment in certification, fair compensation, and advancement infrastructure is simultaneously an investment in reducing intergenerational economic inequality. As the life course literature consistently shows, limited early-life educational and economic opportunities constrain long-term workforce participation in ways that reverberate across generations (30, 40, 41). A well-supported CHW workforce represents one concrete point at which that cycle can be interrupted.

### Limitations

Several limitations should be considered when interpreting these findings. First, participants were recruited from a single CHW training program in Maryland, which may limit transferability to CHW populations trained in other states, programs, or organizational contexts. The specific structure, duration, and support mechanisms of Central Maryland AHEC's program may differ from programs elsewhere, and findings may not generalize to settings with less structured or less well-resourced CHW training infrastructure.

Second, the study used a convenience sample of the first 30 respondents to an invitation email, corresponding to a response rate of approximately 27.5% (30 of 109 eligible CHWs). This approach, while common in qualitative research, may have introduced self-selection bias: respondents may hold more positive views of CHW work and certification, and those with more adverse experiences may be underrepresented. Because only those who responded quickly to a single invitation were enrolled, it is also possible that early responders were disproportionately CHWs who were more professionally engaged or more successful in their careers, which could inflate the positive “certification as a pathway to advancement” narrative central to Theme 1 relative to the experience of the broader, non-responding CHW population.

Third, all interviews were conducted virtually via Zoom, which, while practical for a geographically dispersed sample, may have constrained the depth of engagement compared to in-person interviews.

Fourth, although multiple strategies were employed to enhance trustworthiness, the reflexive thematic analysis approach is inherently interpretive, and alternative analytical framings of the same data are possible. Relatedly, all interviews were conducted by a single CHW interviewer who shared professional identity with participants; while this was intended to build rapport, it may also have encouraged socially desirable responses or discouraged candid criticism of certification and of the CHW role, since participants were speaking with a peer/community member rather than an outside researcher.

Fifth, the study did not include perspectives from CHW supervisors, employers, or healthcare system administrators, whose perspectives might provide additional context for understanding the structural conditions CHWs navigate. Finally, excluding non-certified CHWs limits direct comparison of how certification shapes workforce experiences relative to those without credentials. Future research incorporating both certified and non-certified CHWs, as well as employer and administrator perspectives, would strengthen the understanding of certification's unique contributions to career advancement.

## Conclusion

This study demonstrates that CHW certification functions as more than a professional credential; it operates as a mechanism for career development, identity formation, and long-term workforce retention. For certified CHWs in Maryland, certification enabled career transitions, motivated further education, and sustained commitment to public health service. Participants’ accounts of CHW work as a calling rooted in advocacy, community empowerment, and bridging gaps that clinical systems fail to address point to a workforce that is deeply motivated yet structurally underinvested. Realizing that potential, however, requires confronting the structural failures the data make plain: inadequate compensation, precarious employment, institutional under-recognition, and insufficient career advancement infrastructure. A workforce sustained by passion alone is fragile. Policymakers, health systems, and CHW training programmes must move beyond credentialing and invest in conditions, fair wages, sustainable employment, clear scope-of-practice frameworks, continuing education, and mentorship that allow CHWs to build careers, not just jobs. Doing so will strengthen not only the CHW workforce but the broader effort to build a more equitable and resilient public health system.

These findings suggest that certification alone is insufficient without broader investment in the CHW workforce. Fair compensation, stable employment, and clear advancement pathways are needed if certification is to translate into meaningful professional mobility. Participants’ experiences also point to the importance of training beyond initial certification, including continuing education, mentorship, and stackable credentials that can support movement into supervisory and public health leadership roles. At the organizational level, clearer orientation for supervisors and clinical staff may reduce role ambiguity, improve interdisciplinary collaboration, and ensure CHW contributions are recognized and used effectively. The barriers participants described, including practicum placement challenges, administrative delays, caregiving responsibilities, and health concerns, further underscore the need for flexible and accessible certification models. Future research should include non-certified CHWs, employers, administrators, and policymakers, using longitudinal, multi-stakeholder designs to examine whether certification improves career outcomes and community health.

## Data Availability

The original contributions presented in the study are included in the article/SuppFlementary Material, further inquiries can be directed to the corresponding author.
