# Supplementary material for: Community health worker certification as a pathway to workforce development and career advancement: a qualitative study
Source: Front Health Serv. 2026 Oct 5;6:1902919. doi: 10.3389/frhs.2026.1902919 (PMC13677856; doi:10.3389/frhs.2026.1902919)
Supplement: Supplementary file 1 [file Datasheet1.pdf]

COMMUNITY HEALTH WORKER (CHW) CERTIFICATION INTERVIEW GUIDE

Section A: Background and Previous Employment

1. Employment Before CHW Training

- What job were you doing before you completed your Community Health Worker (CHW) training?
- Can you tell me about your previous work experience before becoming a CHW?

2. Previous Work Experience

- How did your previous work experience influence your decision to become a CHW?
- Did any of your previous work experience prepare you for your current role as a CHW? If yes, how?

Section B: CHW Training and Certification

3. CHW Training

- What motivated you to undertake CHW training?
- Can you describe your experience during the CHW training?
- What were the most important skills or knowledge you gained from the training?

4. Certification

- How long after completing your CHW training did you receive your certification?
- What challenges, if any, did you experience during the certification process?
- What factors helped or supported you in obtaining your CHW certification?

Section C: Employment and Career Pathway

5. Employment After Certification

- How many jobs have you had since obtaining your CHW certification?
- What types of jobs have you held since becoming certified?
- How long did it take you to obtain your first job after CHW certification?
- How did your CHW certification influence your ability to obtain employment?

6. Current Employment

- What is your current position?
- How long have you been working in your current position?
- Can you describe your main responsibilities in your current role?

Section D: CHW Role and Experience

7. Experience as a CHW

- What do you enjoy most about being a CHW?
- What does being a CHW mean to you?
- What motivated you to continue working as a CHW?
- Can you describe a particularly meaningful experience you have had as a CHW?

8. CHW Roles and Responsibilities

- What are the main roles and responsibilities you perform as a CHW?
- How do you support and advocate for members of the community?
- How do you connect community members with health and social resources?
- How do you work with other healthcare or community professionals?

Section E: CHW Training and Current Role

9. Application of CHW Training

- Does your CHW training help you carry out your role in your current position? If yes, how?
- What knowledge or skills do you use from your CHW training most frequently in your current work?
- Are there aspects of your current role for which your CHW training did not adequately prepare you?
- What additional training or skills would help you perform your current role more effectively?

Section F: Other Qualifications and Certifications

10. Other Certifications

- What other certifications or professional qualifications do you hold apart from your CHW certification?
- How have these additional certifications influenced your career?

11. Complementarity of Qualifications

- How do you think your CHW certification complements your other certifications or qualifications?
- In what ways have your different certifications and training helped you obtain or perform your current job?
- Do you think having a CHW certification provides an advantage when seeking employment? Why or why not?

Section G: Career Development and Future Aspirations

12. Career Goals

- What are your career goals going forward?
- Are there additional qualifications, certifications, or degrees you would like to obtain?
- Do you see yourself progressing into a supervisory, management, or leadership position?
- How has your experience as a CHW influenced your career aspirations?

13. Future Sector of Employment

- Do you see yourself continuing to work in the public health sector or moving into another sector? Why?
- What type of organization or setting would you ideally like to work in?
- What role do you see CHWs playing in the future of the healthcare and public health system?

Section H: Overall Reflections and Recommendations

14. Overall Experience

- What has been the most rewarding aspect of working as a CHW?
- What are the major challenges you have experienced as a CHW?
- Do you feel that CHWs are adequately recognized and valued in the healthcare system? Why or why not?
- What changes would improve the experience and working conditions of CHWs?

15. Recommendations

- What recommendations would you make to improve CHW training and certification?
- What recommendations would you make to improve employment opportunities for CHWs?
- What support do you think CHWs need to advance their careers?

16. Final Question

- Is there anything else you would like to share about your experience as a CHW that we have not discussed?
- Do you have any additional thoughts or recommendations regarding CHW training, certification, employment, or career development?
